# (*N* ^4^-*n*-Butyl­pyridine-4-carbothio­amide-κ*N* ^4^)chloridobis(dimethyl­glyoximato-κ^2^ *N*,*N*′)cobalt(III) hemihydrate

**DOI:** 10.1107/S1600536809022661

**Published:** 2009-06-20

**Authors:** C. Revathi, A. Dayalan, K. SethuSankar

**Affiliations:** a Loyola College (Autonomous), Chennai 600 034, Tamil Nadu, India; b RKM Vivekananda College (Autonomous), Chennai 600 004, Tamil Nadu, India

## Abstract

The title compound, *trans*-[Co(C_4_H_7_N_2_O_2_)_2_Cl(C_10_H_14_N_2_S)]·0.5H_2_O, contains two independent mol­ecules in the asymmetric unit in which the Co^III^ ions are coordinated in slightly distorted octa­hedral coordination environments. The bis-chelating glyoximate ligands, which occupy equatorial sites, are linked by interligand O—H⋯O hydrogen bonds. The dihedral angles between the mean planes of the glyoximate ligands in each mol­ecule are 2.07 (8) and 1.60 (1)°. The asymmetric unit contains a solvent water mol­ecule which is disordered over two sites with refined occupancies 0.64 (2) and 0.36 (2).

## Related literature

For a related structure, see: Kavitha *et al.* (2008 ▶). For background, see: Trogler *et al.* (1974 ▶); Dolphin (1982 ▶); Bresciani-Pahor *et al.* (1985 ▶); Geno & Halpern (1987 ▶); Englert *et al.* (1999 ▶, 2000 ▶). For the synthetic prodedure, see: Schrauzer & Kohnel (1964 ▶); Ramesh *et al.* (2008 ▶).
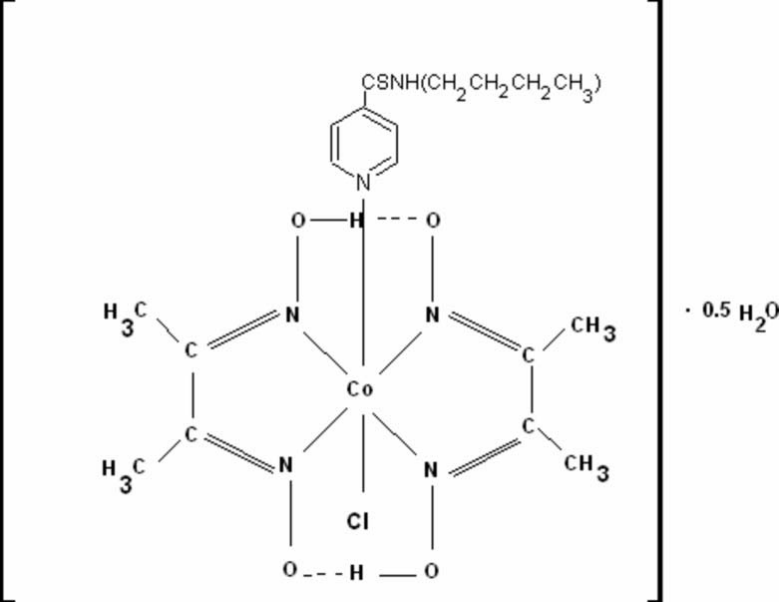


## Experimental

### 

#### Crystal data

[Co(C_4_H_7_N_2_O_2_)_2_Cl(C_10_H_14_N_2_S)]·0.5H_2_O
*M* *_r_* = 526.90
Monoclinic, 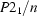

*a* = 11.1976 (5) Å
*b* = 14.7889 (7) Å
*c* = 28.8482 (14) Å
β = 95.748 (2)°
*V* = 4753.2 (4) Å^3^
*Z* = 8
Mo *K*α radiation
μ = 0.96 mm^−1^
*T* = 293 K
0.3 × 0.2 × 0.2 mm

#### Data collection

Bruker Kappa APEXII diffractometer
Absorption correction: multi-scan (*SADABS*; Bruker, 2004 ▶) *T*_min_ = 0.742, *T*_max_ = 0.853
50767 measured reflections
10297 independent reflections
7510 reflections with *I* > 2σ(*I*)
*R*_int_ = 0.044

#### Refinement

*R*[*F* ^2^ > 2σ(*F* ^2^)] = 0.053
*wR*(*F* ^2^) = 0.168
*S* = 1.06
10297 reflections
606 parameters
1 restraint
H atoms treated by a mixture of independent and constrained refinement
Δρ_max_ = 0.96 e Å^−3^
Δρ_min_ = −0.82 e Å^−3^

### 

Data collection: *APEX2* (Bruker, 2004 ▶); cell refinement: *APEX2* and *SAINT* (Bruker, 2004 ▶); data reduction: *SAINT*; program(s) used to solve structure: *SIR92* (Altomare *et al*., 1993 ▶); program(s) used to refine structure: *SHELXL97* (Sheldrick, 2008 ▶); molecular graphics: *PLATON* (Spek, 2009 ▶); software used to prepare material for publication: *SHELXL97*.

## Supplementary Material

Crystal structure: contains datablocks global, I. DOI: 10.1107/S1600536809022661/lh2818sup1.cif

Structure factors: contains datablocks I. DOI: 10.1107/S1600536809022661/lh2818Isup2.hkl

Additional supplementary materials:  crystallographic information; 3D view; checkCIF report

**Fig. 1.**
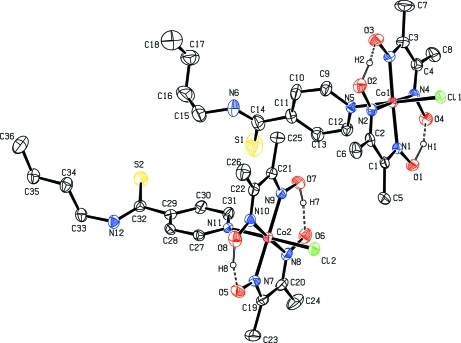
The asymmetric unit with 30% probability ellipsoids. Hydrogen atoms and the solvent water is omitted for clarity. Dashed lines indicate hydrogen bonds.

Enhanced figure: interactive version of Fig. 1

## Figures and Tables

**Table 1:** Hydrogen-bond geometry (Å, °)

*D*—H⋯*A*	*D*—H	H⋯*A*	*D*⋯*A*	*D*—H⋯*A*
O1—H1⋯O4	1.20 (6)	1.32 (6)	2.491 (4)	162 (5)
O2—H2⋯O3	1.12 (6)	1.39 (6)	2.469 (4)	158 (5)
O8—H8⋯O5	1.17 (8)	1.34 (8)	2.486 (5)	165 (6)
O7—H7⋯O6	0.98 (7)	1.52 (7)	2.490 (5)	171 (7)

## supplementary crystallographic information

### Comment

The study of simple models of the B_12_ coenzyme such as the cobaloximes, [RCo(dmgH)_2_L] where *R*= alkyl group, dmgH^-^= dimethylglyoximate and *L*=neutral ligand, has furnished significant amounts of data that have provided a foundation for understanding the behaviour of cobaloximes (Trogler *et al.*, 1974). Compared to other cobalamins and other model systems, cobaloximes have shorter Co-*L* bonds where *L*= pyridine or a substituted pyridine group. It is known that such a metal coenzyme is related to a number of 1,2-intramolecular rearangement enzymatic reactions (Dolphin *et al.*, 1982). Early X-ray diffraction analysis has shown that the coenzyme has a bulky corrin ring in the equatorial position (Bresciani-Pahor *et al.*, 1985) the deadenosyl group and 5,6-dimethylbenzimidazole group as the axial ligands. The flexiblity of the equatorial oxime ligands is quite similar to that of corrin in neutral co-factor (Geno & Halpern, 1987). In the title compond the coordination about the Co^III^ ion is slightly distorted octahedral with the  the N-n-Butyl-4-pyridinecarbothioamide and chloride ligands occupy the axial positions and the two dimethyl glyoximato ligands occupy the equatorial sites. The axial bonds are essentially perpendicular (see coordination bond angles) to the equatorial glyoximate least-squares planes (with maximum deviation from the planes of 0.054 (2) and 0.072 (2)Å for O3 and O8 respectively). In one molecule the *n*-butyl group is in an extended conformation, while in the other it has a coiled conformation as described by the torsion angles N12-C33-C34-C35 = 177.7 (4) and N6-C15-C16-C17 = -51.0 (4) °, respectively. The dihedral angle between the mean planes of the glyoximato ligands in each molecule are 2.07 (8)° and 1.60 (1)° (cf. Englert *et al.*, 1999;2000). There is one weak C-H···Cl interaction in involving chlorine atom of one molecule and an H atom from the other independent molecule [H28···Cl1^i^ = 2.72Å; C28-H28-Cl1^i^ = 156° and C28···Cl1^i^ = 3.591 (4)Å; symmetry code (i): 3/2-x, -1/2+y, 1/2-z].

### Experimental

The title compound was synthesized by a literature method (Schrauzer & Kohnel, 1964), using H[Co(dmgH)_2_Cl_2_] as the starting material (Ramesh *et al.*, 2008). The dichloro cobaloxime was mixed with N-n-Bu-4-PCT in 1:1 molar ratio in about 60 ml of absolute ethanol and allowed to stir for 3hrs with warming. The resulting brown coloured complex was filtered and washed with absolute ethanol and ether and dried over vacuum desiccator. Crystals of the complex were grown in ethanol by slow evaporation. The purity of the complex was ascertained by UV-Vis, IR and NMR.

### Refinement

H atoms were visible in difference Fourier maps but those bonded to H atoms were placed idealized positions and included in the refinement in a riding-model approximation with C—H(aromatic) = 0.93Å and U_iso_(H) = 1.2*U*_eq_C; C—H(methyl) = 0.96A%, and U_iso_(H) = 1.5*U*_eq_C.A%. H atoms bonded to O atoms in the complex molecules were refined independently with isotropic displacement parameters. The H atoms of the disordered water atoms were not located and are not included in the refinement but are however included in the molecular formula. They were not considered in the hydrogen bonding motif.

### Crystal data

**Table:**

[Co(C_4_H_7_N_2_O_2_)_2_Cl(C_10_H_14_N_2_S)]·0.5H_2_O	*F*(000) = 2192
*M**_r_* = 526.90	*D*_x_ = 1.473 Mg m^−^^3^
Monoclinic, *P*2_1_/*n*	Mo *K*α radiation, λ = 0.71073 Å
Hall symbol: -P 2yn	Cell parameters from 5420 reflections
*a* = 11.1976 (5) Å	θ = 2.5–26.0°
*b* = 14.7889 (7) Å	µ = 0.96 mm^−^^1^
*c* = 28.8482 (14) Å	*T* = 293 K
β = 95.748 (2)°	Needle, brown
*V* = 4753.2 (4) Å^3^	0.3 × 0.2 × 0.2 mm
*Z* = 8	

### Data collection

**Table:**

Bruker Kappa-APEX2 diffractometer	10297 independent reflections
Radiation source: fine-focus sealed tube	7510 reflections with *I* > 2σ(*I*)
graphite	*R*_int_ = 0.044
Detector resolution: 0 pixels mm^-1^	θ_max_ = 26.9°, θ_min_ = 1.4°
ω and φ scans	*h* = −14→10
Absorption correction: multi-scan (*SADABS*; Bruker, 2004)	*k* = −18→18
*T*_min_ = 0.742, *T*_max_ = 0.853	*l* = −36→36
50767 measured reflections	

### Refinement

**Table:**

Refinement on *F*^2^	Secondary atom site location: difference Fourier map
Least-squares matrix: full	Hydrogen site location: inferred from neighbouring sites
*R*[*F*^2^ > 2σ(*F*^2^)] = 0.053	H atoms treated by a mixture of independent and constrained refinement
*wR*(*F*^2^) = 0.168	*w* = 1/[σ^2^(*F*_o_^2^) + (0.0927*P*)^2^ + 3.9112*P*] where *P* = (*F*_o_^2^ + 2*F*_c_^2^)/3
*S* = 1.06	(Δ/σ)_max_ = 0.009
10297 reflections	Δρ_max_ = 0.96 e Å^−^^3^
606 parameters	Δρ_min_ = −0.82 e Å^−^^3^
1 restraint	Extinction correction: *SHELXL97* (Sheldrick, 2008), Fc^*^=kFc[1+0.001xFc^2^λ^3^/sin(2θ)]^-1/4^
Primary atom site location: structure-invariant direct methods	Extinction coefficient: 0.0023 (3)

### Special details

**Table:**

Geometry. All e.s.d.'s (except the e.s.d. in the dihedral angle between two l.s. planes) are estimated using the full covariance matrix. The cell e.s.d.'s are taken into account individually in the estimation of e.s.d.'s in distances, angles and torsion angles; correlations between e.s.d.'s in cell parameters are only used when they are defined by crystal symmetry. An approximate (isotropic) treatment of cell e.s.d.'s is used for estimating e.s.d.'s involving l.s. planes.
Refinement. Refinement of *F*^2^ against ALL reflections. The weighted *R*-factor *wR* and goodness of fit *S* are based on *F*^2^, conventional *R*-factors *R* are based on *F*, with *F* set to zero for negative *F*^2^. The threshold expression of *F*^2^ > σ(*F*^2^) is used only for calculating *R*-factors(gt) *etc*. and is not relevant to the choice of reflections for refinement. *R*-factors based on *F*^2^ are statistically about twice as large as those based on *F*, and *R*- factors based on ALL data will be even larger.

### Fractional atomic coordinates and isotropic or equivalent isotropic displacement parameters (Å^2^)

**Table:**

	*x*	*y*	*z*	*U*_iso_*/*U*_eq_	Occ. (<1)
C1	0.6540 (3)	0.4012 (2)	0.14607 (12)	0.0391 (8)	
C2	0.5817 (3)	0.3183 (3)	0.13929 (13)	0.0395 (8)	
C3	0.7787 (4)	0.2495 (3)	−0.01437 (14)	0.0480 (9)	
C4	0.8518 (3)	0.3305 (3)	−0.00758 (13)	0.0432 (9)	
C5	0.6493 (4)	0.4620 (3)	0.18669 (13)	0.0548 (11)	
H5A	0.6895	0.5177	0.1811	0.082*	
H5B	0.6884	0.4335	0.2140	0.082*	
H5C	0.5671	0.4742	0.1913	0.082*	
C6	0.4897 (4)	0.2917 (3)	0.17091 (16)	0.0579 (12)	
H6A	0.4253	0.3348	0.1682	0.087*	
H6B	0.5259	0.2903	0.2025	0.087*	
H6C	0.4587	0.2329	0.1623	0.087*	
C7	0.7738 (6)	0.1895 (4)	−0.05624 (19)	0.091 (2)	
H7A	0.7375	0.1328	−0.0494	0.137*	
H7B	0.8537	0.1790	−0.0644	0.137*	
H7C	0.7270	0.2180	−0.0818	0.137*	
C8	0.9383 (4)	0.3614 (3)	−0.04059 (15)	0.0570 (11)	
H8A	0.9657	0.4213	−0.0323	0.085*	
H8B	0.8991	0.3618	−0.0717	0.085*	
H8C	1.0056	0.3209	−0.0389	0.085*	
C9	0.8419 (4)	0.1681 (3)	0.10754 (17)	0.0542 (11)	
H9	0.7787	0.1375	0.0911	0.065*	
C10	0.9260 (4)	0.1193 (3)	0.13510 (17)	0.0587 (12)	
H10	0.9197	0.0567	0.1368	0.070*	
C11	1.0198 (4)	0.1636 (3)	0.16024 (14)	0.0486 (10)	
C12	1.0274 (4)	0.2554 (3)	0.15450 (16)	0.0536 (10)	
H12	1.0911	0.2873	0.1698	0.064*	
C13	0.9412 (4)	0.3003 (3)	0.12621 (14)	0.0458 (9)	
H13	0.9481	0.3626	0.1227	0.055*	
C14	1.1118 (4)	0.1146 (4)	0.19326 (17)	0.0641 (12)	
C15	1.1459 (6)	−0.0284 (5)	0.2383 (2)	0.0906 (19)	
H15A	1.1778	0.0094	0.2641	0.109*	
H15B	1.0891	−0.0699	0.2501	0.109*	
C16	1.2409 (7)	−0.0793 (5)	0.2233 (2)	0.113 (3)	
H16A	1.2700	−0.1194	0.2485	0.136*	
H16B	1.3056	−0.0376	0.2186	0.136*	
C17	1.2186 (6)	−0.1359 (5)	0.1797 (2)	0.0895 (18)	
H17A	1.1807	−0.0978	0.1551	0.107*	
H17B	1.2957	−0.1545	0.1704	0.107*	
C18	1.1436 (6)	−0.2176 (5)	0.1829 (3)	0.107 (2)	
H18A	1.1685	−0.2491	0.2114	0.161*	
H18B	1.1528	−0.2564	0.1569	0.161*	
H18C	1.0610	−0.2002	0.1827	0.161*	
C19	0.8873 (4)	0.4019 (3)	0.40245 (15)	0.0548 (11)	
C20	0.9481 (4)	0.4264 (3)	0.36220 (16)	0.0510 (10)	
C21	0.7077 (4)	0.1755 (3)	0.26363 (13)	0.0442 (9)	
C22	0.6402 (3)	0.1546 (3)	0.30342 (14)	0.0441 (9)	
C23	0.9121 (6)	0.4454 (4)	0.44954 (18)	0.0843 (18)	
H23A	0.8644	0.4167	0.4712	0.126*	
H23B	0.8920	0.5085	0.4473	0.126*	
H23C	0.9956	0.4389	0.4602	0.126*	
C24	1.0394 (5)	0.4994 (3)	0.3627 (2)	0.0781 (16)	
H24A	1.0620	0.5080	0.3317	0.117*	
H24B	1.1088	0.4828	0.3831	0.117*	
H24C	1.0063	0.5545	0.3734	0.117*	
C25	0.6947 (5)	0.1242 (3)	0.21891 (16)	0.0650 (13)	
H25A	0.7317	0.1575	0.1956	0.098*	
H25B	0.6111	0.1157	0.2089	0.098*	
H25C	0.7330	0.0663	0.2234	0.098*	
C26	0.5420 (4)	0.0867 (3)	0.3009 (2)	0.0681 (13)	
H26A	0.5214	0.0740	0.3317	0.102*	
H26B	0.5682	0.0321	0.2871	0.102*	
H26C	0.4731	0.1102	0.2823	0.102*	
C27	0.9110 (3)	0.1502 (2)	0.38996 (12)	0.0374 (8)	
H27	0.8439	0.1560	0.4063	0.045*	
C28	0.9965 (3)	0.0876 (3)	0.40509 (12)	0.0375 (8)	
H28	0.9862	0.0517	0.4309	0.045*	
C29	1.0991 (3)	0.0776 (2)	0.38179 (12)	0.0361 (8)	
C30	1.1068 (4)	0.1332 (3)	0.34363 (14)	0.0447 (9)	
H30	1.1735	0.1293	0.3270	0.054*	
C31	1.0177 (4)	0.1937 (3)	0.33005 (14)	0.0430 (9)	
H31	1.0254	0.2296	0.3040	0.052*	
C32	1.1937 (3)	0.0082 (3)	0.39541 (13)	0.0397 (8)	
C33	1.2757 (4)	−0.0957 (3)	0.45690 (15)	0.0512 (10)	
H33A	1.2829	−0.0937	0.4907	0.061*	
H33B	1.3549	−0.0859	0.4469	0.061*	
C34	1.2308 (4)	−0.1880 (3)	0.44078 (18)	0.0555 (11)	
H34A	1.1529	−0.1982	0.4518	0.067*	
H34B	1.2202	−0.1887	0.4070	0.067*	
C35	1.3131 (4)	−0.2642 (3)	0.45739 (17)	0.0588 (11)	
H35A	1.3197	−0.2665	0.4912	0.071*	
H35B	1.3925	−0.2527	0.4480	0.071*	
C36	1.2683 (5)	−0.3549 (4)	0.4380 (2)	0.0869 (18)	
H36A	1.1937	−0.3695	0.4499	0.130*	
H36B	1.3265	−0.4008	0.4471	0.130*	
H36C	1.2564	−0.3516	0.4046	0.130*	
N1	0.7222 (3)	0.4142 (2)	0.11309 (10)	0.0356 (6)	
N2	0.6040 (3)	0.2735 (2)	0.10299 (10)	0.0359 (6)	
N3	0.7116 (3)	0.2353 (2)	0.01918 (11)	0.0403 (7)	
N4	0.8335 (3)	0.3740 (2)	0.02985 (10)	0.0355 (6)	
N5	0.8476 (3)	0.2575 (2)	0.10354 (10)	0.0347 (6)	
N6	1.0805 (4)	0.0297 (3)	0.20319 (16)	0.0767 (13)	
N7	0.8069 (3)	0.3394 (2)	0.39383 (11)	0.0465 (8)	
N8	0.9133 (3)	0.3797 (2)	0.32561 (12)	0.0444 (8)	
N9	0.7812 (3)	0.2424 (2)	0.27193 (10)	0.0388 (7)	
N10	0.6723 (3)	0.2043 (2)	0.33924 (11)	0.0409 (7)	
N11	0.9195 (3)	0.20336 (19)	0.35282 (10)	0.0331 (6)	
N12	1.1953 (3)	−0.0228 (2)	0.43840 (12)	0.0465 (8)	
O1	0.7913 (3)	0.48767 (17)	0.11270 (9)	0.0465 (6)	
O2	0.5463 (3)	0.19640 (18)	0.09123 (11)	0.0504 (7)	
O3	0.6361 (3)	0.1655 (2)	0.01808 (11)	0.0589 (8)	
O4	0.8906 (2)	0.45087 (19)	0.04153 (10)	0.0470 (6)	
O5	0.7379 (3)	0.3099 (2)	0.42581 (10)	0.0642 (9)	
O6	0.9561 (3)	0.3945 (2)	0.28445 (11)	0.0569 (8)	
O7	0.8475 (3)	0.2714 (2)	0.23890 (10)	0.0545 (7)	
O8	0.6170 (3)	0.1955 (2)	0.37864 (11)	0.0570 (8)	
O9	0.5206 (13)	0.4306 (11)	0.4152 (10)	0.117 (9)	0.36 (2)
O9'	0.563 (2)	0.4610 (14)	0.4517 (9)	0.258 (10)	0.64 (2)
S1	1.22943 (19)	0.16153 (15)	0.21468 (9)	0.1222 (8)	
S2	1.28739 (11)	−0.02367 (9)	0.35816 (4)	0.0642 (3)	
Cl1	0.57535 (8)	0.39973 (6)	0.02391 (3)	0.0401 (2)	
Cl2	0.65579 (9)	0.39511 (7)	0.31055 (4)	0.0494 (3)	
Co1	0.71972 (4)	0.32373 (3)	0.066535 (15)	0.02991 (14)	
Co2	0.79476 (4)	0.29152 (3)	0.333079 (16)	0.03392 (14)	
H1	0.829 (5)	0.480 (4)	0.076 (2)	0.092 (17)*	
H2	0.566 (5)	0.181 (4)	0.055 (2)	0.090 (18)*	
H8	0.670 (6)	0.244 (5)	0.406 (3)	0.13 (2)*	
H7	0.890 (6)	0.323 (5)	0.254 (3)	0.13 (3)*	

### Atomic displacement parameters (Å^2^)

**Table:**

	*U*^11^	*U*^22^	*U*^33^	*U*^12^	*U*^13^	*U*^23^
C1	0.051 (2)	0.039 (2)	0.0281 (17)	0.0090 (16)	0.0051 (15)	0.0082 (15)
C2	0.0390 (19)	0.046 (2)	0.0340 (19)	0.0050 (16)	0.0060 (15)	0.0135 (16)
C3	0.060 (2)	0.046 (2)	0.039 (2)	0.0048 (19)	0.0079 (18)	−0.0063 (17)
C4	0.044 (2)	0.047 (2)	0.040 (2)	0.0068 (17)	0.0091 (16)	0.0049 (17)
C5	0.077 (3)	0.055 (3)	0.032 (2)	0.011 (2)	0.006 (2)	0.0002 (18)
C6	0.055 (2)	0.071 (3)	0.051 (3)	0.008 (2)	0.020 (2)	0.021 (2)
C7	0.138 (6)	0.084 (4)	0.056 (3)	−0.013 (4)	0.031 (3)	−0.029 (3)
C8	0.055 (2)	0.076 (3)	0.043 (2)	0.004 (2)	0.0205 (19)	0.004 (2)
C9	0.054 (2)	0.039 (2)	0.067 (3)	−0.0056 (19)	−0.009 (2)	0.010 (2)
C10	0.059 (3)	0.039 (2)	0.075 (3)	−0.0016 (19)	−0.008 (2)	0.018 (2)
C11	0.046 (2)	0.056 (3)	0.043 (2)	0.0080 (19)	0.0039 (17)	0.0119 (19)
C12	0.048 (2)	0.053 (3)	0.057 (3)	−0.0051 (19)	−0.0082 (19)	0.005 (2)
C13	0.046 (2)	0.039 (2)	0.051 (2)	−0.0044 (17)	−0.0002 (18)	0.0059 (18)
C14	0.062 (3)	0.073 (3)	0.056 (3)	0.011 (2)	−0.002 (2)	0.009 (2)
C15	0.114 (5)	0.095 (5)	0.060 (3)	0.035 (4)	−0.003 (3)	0.008 (3)
C16	0.132 (6)	0.127 (6)	0.073 (4)	0.057 (5)	−0.023 (4)	−0.020 (4)
C17	0.090 (4)	0.094 (5)	0.083 (4)	0.018 (4)	0.000 (3)	−0.022 (4)
C18	0.077 (4)	0.123 (6)	0.123 (6)	−0.002 (4)	0.016 (4)	−0.013 (5)
C19	0.070 (3)	0.041 (2)	0.049 (2)	0.020 (2)	−0.015 (2)	−0.0140 (19)
C20	0.050 (2)	0.0300 (19)	0.070 (3)	0.0063 (17)	−0.010 (2)	−0.0070 (19)
C21	0.053 (2)	0.039 (2)	0.039 (2)	0.0093 (18)	−0.0025 (17)	−0.0019 (16)
C22	0.042 (2)	0.037 (2)	0.052 (2)	0.0029 (16)	0.0001 (17)	0.0060 (18)
C23	0.116 (5)	0.067 (3)	0.062 (3)	0.021 (3)	−0.029 (3)	−0.028 (3)
C24	0.069 (3)	0.042 (3)	0.120 (5)	−0.004 (2)	−0.008 (3)	−0.015 (3)
C25	0.091 (4)	0.054 (3)	0.047 (3)	0.004 (2)	−0.006 (2)	−0.014 (2)
C26	0.058 (3)	0.059 (3)	0.086 (4)	−0.012 (2)	−0.002 (2)	0.008 (3)
C27	0.0402 (18)	0.042 (2)	0.0309 (18)	0.0037 (16)	0.0081 (14)	0.0000 (15)
C28	0.0417 (19)	0.042 (2)	0.0288 (17)	0.0010 (16)	0.0030 (14)	0.0023 (15)
C29	0.0375 (18)	0.0343 (18)	0.0368 (18)	0.0001 (15)	0.0051 (14)	−0.0064 (15)
C30	0.045 (2)	0.040 (2)	0.052 (2)	0.0029 (17)	0.0173 (17)	0.0030 (18)
C31	0.050 (2)	0.0361 (19)	0.045 (2)	0.0023 (16)	0.0187 (17)	0.0075 (16)
C32	0.0373 (18)	0.0371 (19)	0.044 (2)	−0.0007 (15)	−0.0009 (15)	−0.0043 (16)
C33	0.048 (2)	0.054 (2)	0.049 (2)	0.0111 (19)	−0.0052 (18)	0.004 (2)
C34	0.046 (2)	0.051 (2)	0.069 (3)	0.0056 (19)	−0.002 (2)	0.007 (2)
C35	0.058 (3)	0.056 (3)	0.062 (3)	0.007 (2)	0.000 (2)	0.009 (2)
C36	0.088 (4)	0.051 (3)	0.119 (5)	0.007 (3)	0.000 (4)	0.001 (3)
N1	0.0434 (16)	0.0311 (15)	0.0319 (15)	−0.0013 (12)	0.0025 (12)	0.0033 (12)
N2	0.0349 (15)	0.0338 (15)	0.0393 (16)	−0.0028 (12)	0.0060 (12)	0.0079 (13)
N3	0.0486 (17)	0.0327 (16)	0.0391 (17)	−0.0030 (13)	0.0017 (14)	−0.0028 (13)
N4	0.0390 (15)	0.0349 (16)	0.0333 (15)	−0.0028 (13)	0.0069 (12)	0.0044 (12)
N5	0.0363 (15)	0.0330 (15)	0.0353 (15)	−0.0023 (12)	0.0066 (12)	0.0036 (12)
N6	0.092 (3)	0.061 (3)	0.074 (3)	0.022 (2)	−0.009 (2)	0.017 (2)
N7	0.062 (2)	0.0415 (19)	0.0356 (17)	0.0155 (16)	0.0042 (15)	−0.0054 (14)
N8	0.0500 (18)	0.0311 (16)	0.0513 (19)	0.0065 (14)	0.0022 (15)	0.0021 (14)
N9	0.0497 (17)	0.0362 (16)	0.0315 (15)	0.0042 (14)	0.0088 (13)	0.0021 (13)
N10	0.0433 (17)	0.0425 (17)	0.0382 (17)	0.0051 (14)	0.0099 (13)	0.0057 (14)
N11	0.0403 (15)	0.0281 (14)	0.0313 (15)	0.0018 (12)	0.0053 (12)	−0.0008 (12)
N12	0.0441 (17)	0.0459 (19)	0.0483 (19)	0.0084 (15)	−0.0002 (14)	0.0053 (15)
O1	0.0613 (17)	0.0344 (14)	0.0440 (15)	−0.0121 (12)	0.0056 (13)	−0.0032 (12)
O2	0.0487 (16)	0.0403 (15)	0.0625 (19)	−0.0163 (12)	0.0079 (14)	0.0053 (13)
O3	0.073 (2)	0.0419 (16)	0.0602 (19)	−0.0208 (15)	0.0010 (16)	−0.0103 (14)
O4	0.0496 (15)	0.0447 (15)	0.0475 (15)	−0.0196 (13)	0.0094 (12)	0.0037 (12)
O5	0.089 (2)	0.070 (2)	0.0364 (16)	0.0175 (18)	0.0160 (16)	−0.0037 (15)
O6	0.0630 (18)	0.0475 (17)	0.0631 (19)	−0.0028 (14)	0.0211 (15)	0.0125 (14)
O7	0.074 (2)	0.0575 (19)	0.0352 (15)	0.0007 (16)	0.0202 (14)	0.0040 (13)
O8	0.0560 (17)	0.068 (2)	0.0516 (18)	0.0074 (15)	0.0273 (14)	0.0159 (15)
O9	0.071 (9)	0.089 (10)	0.20 (2)	0.009 (7)	0.048 (10)	−0.003 (11)
O9'	0.34 (2)	0.181 (15)	0.24 (2)	0.073 (15)	−0.035 (18)	0.031 (15)
S1	0.1044 (13)	0.1075 (15)	0.1419 (18)	−0.0176 (11)	−0.0507 (13)	0.0240 (13)
S2	0.0658 (7)	0.0746 (8)	0.0535 (7)	0.0232 (6)	0.0127 (6)	0.0032 (6)
Cl1	0.0417 (5)	0.0443 (5)	0.0342 (4)	0.0051 (4)	0.0029 (3)	0.0077 (4)
Cl2	0.0552 (6)	0.0440 (5)	0.0482 (5)	0.0141 (4)	0.0019 (4)	0.0042 (4)
Co1	0.0343 (2)	0.0272 (2)	0.0284 (2)	−0.00359 (18)	0.00409 (18)	0.00201 (18)
Co2	0.0419 (3)	0.0305 (3)	0.0297 (3)	0.0035 (2)	0.00545 (19)	0.00001 (19)

### Geometric parameters (Å, °)

**Table:**

C1—N1	1.293 (4)	C24—H24B	0.9600
C1—C2	1.471 (5)	C24—H24C	0.9600
C1—C5	1.483 (5)	C25—H25A	0.9600
C2—N2	1.284 (5)	C25—H25B	0.9600
C2—C6	1.496 (5)	C25—H25C	0.9600
C3—N3	1.300 (5)	C26—H26A	0.9600
C3—C4	1.453 (6)	C26—H26B	0.9600
C3—C7	1.495 (6)	C26—H26C	0.9600
C4—N4	1.291 (5)	C27—N11	1.340 (4)
C4—C8	1.496 (5)	C27—C28	1.371 (5)
C5—H5A	0.9600	C27—H27	0.9300
C5—H5B	0.9600	C28—C29	1.395 (5)
C5—H5C	0.9600	C28—H28	0.9300
C6—H6A	0.9600	C29—C30	1.383 (5)
C6—H6B	0.9600	C29—C32	1.499 (5)
C6—H6C	0.9600	C30—C31	1.368 (5)
C7—H7A	0.9600	C30—H30	0.9300
C7—H7B	0.9600	C31—N11	1.343 (5)
C7—H7C	0.9600	C31—H31	0.9300
C8—H8A	0.9600	C32—N12	1.321 (5)
C8—H8B	0.9600	C32—S2	1.644 (4)
C8—H8C	0.9600	C33—N12	1.469 (5)
C9—N5	1.330 (5)	C33—C34	1.512 (6)
C9—C10	1.374 (6)	C33—H33A	0.9700
C9—H9	0.9300	C33—H33B	0.9700
C10—C11	1.381 (6)	C34—C35	1.503 (6)
C10—H10	0.9300	C34—H34A	0.9700
C11—C12	1.371 (6)	C34—H34B	0.9700
C11—C14	1.516 (6)	C35—C36	1.519 (7)
C12—C13	1.371 (6)	C35—H35A	0.9700
C12—H12	0.9300	C35—H35B	0.9700
C13—N5	1.337 (5)	C36—H36A	0.9600
C13—H13	0.9300	C36—H36B	0.9600
C14—N6	1.343 (7)	C36—H36C	0.9600
C14—S1	1.560 (5)	N1—O1	1.335 (4)
C15—C16	1.407 (8)	N1—Co1	1.894 (3)
C15—N6	1.467 (6)	N2—O2	1.338 (4)
C15—H15A	0.9700	N2—Co1	1.900 (3)
C15—H15B	0.9700	N3—O3	1.333 (4)
C16—C17	1.510 (8)	N3—Co1	1.887 (3)
C16—H16A	0.9700	N4—O4	1.331 (4)
C16—H16B	0.9700	N4—Co1	1.889 (3)
C17—C18	1.480 (9)	N5—Co1	1.960 (3)
C17—H17A	0.9700	N7—O5	1.334 (5)
C17—H17B	0.9700	N7—Co2	1.882 (3)
C18—H18A	0.9600	N8—O6	1.343 (4)
C18—H18B	0.9600	N8—Co2	1.888 (3)
C18—H18C	0.9600	N9—O7	1.336 (4)
C19—N7	1.297 (6)	N9—Co2	1.900 (3)
C19—C20	1.450 (7)	N10—O8	1.354 (4)
C19—C23	1.504 (6)	N10—Co2	1.904 (3)
C20—N8	1.288 (5)	N11—Co2	1.954 (3)
C20—C24	1.486 (6)	O1—H1	1.20 (6)
C21—N9	1.293 (5)	O2—H2	1.12 (6)
C21—C22	1.469 (6)	O3—H2	1.39 (6)
C21—C25	1.492 (6)	O4—H1	1.32 (6)
C22—N10	1.290 (5)	O5—H8	1.34 (8)
C22—C26	1.485 (6)	O7—H7	0.98 (7)
C23—H23A	0.9600	O8—H8	1.17 (8)
C23—H23B	0.9600	Cl1—Co1	2.2341 (10)
C23—H23C	0.9600	Cl2—Co2	2.2334 (10)
C24—H24A	0.9600		
			
N1—C1—C2	112.6 (3)	H26B—C26—H26C	109.5
N1—C1—C5	124.2 (4)	N11—C27—C28	123.2 (3)
C2—C1—C5	123.3 (3)	N11—C27—H27	118.4
N2—C2—C1	112.9 (3)	C28—C27—H27	118.4
N2—C2—C6	124.3 (4)	C27—C28—C29	120.0 (3)
C1—C2—C6	122.8 (4)	C27—C28—H28	120.0
N3—C3—C4	113.1 (3)	C29—C28—H28	120.0
N3—C3—C7	121.9 (4)	C30—C29—C28	116.2 (3)
C4—C3—C7	125.0 (4)	C30—C29—C32	121.1 (3)
N4—C4—C3	112.9 (3)	C28—C29—C32	122.7 (3)
N4—C4—C8	123.2 (4)	C31—C30—C29	121.0 (3)
C3—C4—C8	123.9 (4)	C31—C30—H30	119.5
C1—C5—H5A	109.5	C29—C30—H30	119.5
C1—C5—H5B	109.5	N11—C31—C30	122.6 (3)
H5A—C5—H5B	109.5	N11—C31—H31	118.7
C1—C5—H5C	109.5	C30—C31—H31	118.7
H5A—C5—H5C	109.5	N12—C32—C29	115.3 (3)
H5B—C5—H5C	109.5	N12—C32—S2	124.3 (3)
C2—C6—H6A	109.5	C29—C32—S2	120.4 (3)
C2—C6—H6B	109.5	N12—C33—C34	112.2 (3)
H6A—C6—H6B	109.5	N12—C33—H33A	109.2
C2—C6—H6C	109.5	C34—C33—H33A	109.2
H6A—C6—H6C	109.5	N12—C33—H33B	109.2
H6B—C6—H6C	109.5	C34—C33—H33B	109.2
C3—C7—H7A	109.5	H33A—C33—H33B	107.9
C3—C7—H7B	109.5	C35—C34—C33	113.9 (4)
H7A—C7—H7B	109.5	C35—C34—H34A	108.8
C3—C7—H7C	109.5	C33—C34—H34A	108.8
H7A—C7—H7C	109.5	C35—C34—H34B	108.8
H7B—C7—H7C	109.5	C33—C34—H34B	108.8
C4—C8—H8A	109.5	H34A—C34—H34B	107.7
C4—C8—H8B	109.5	C34—C35—C36	112.0 (4)
H8A—C8—H8B	109.5	C34—C35—H35A	109.2
C4—C8—H8C	109.5	C36—C35—H35A	109.2
H8A—C8—H8C	109.5	C34—C35—H35B	109.2
H8B—C8—H8C	109.5	C36—C35—H35B	109.2
N5—C9—C10	122.4 (4)	H35A—C35—H35B	107.9
N5—C9—H9	118.8	C35—C36—H36A	109.5
C10—C9—H9	118.8	C35—C36—H36B	109.5
C9—C10—C11	119.7 (4)	H36A—C36—H36B	109.5
C9—C10—H10	120.1	C35—C36—H36C	109.5
C11—C10—H10	120.1	H36A—C36—H36C	109.5
C12—C11—C10	117.4 (4)	H36B—C36—H36C	109.5
C12—C11—C14	120.2 (4)	C1—N1—O1	120.9 (3)
C10—C11—C14	122.5 (4)	C1—N1—Co1	116.7 (3)
C13—C12—C11	120.2 (4)	O1—N1—Co1	122.5 (2)
C13—C12—H12	119.9	C2—N2—O2	121.2 (3)
C11—C12—H12	119.9	C2—N2—Co1	116.7 (3)
N5—C13—C12	122.1 (4)	O2—N2—Co1	122.1 (2)
N5—C13—H13	118.9	C3—N3—O3	121.3 (3)
C12—C13—H13	118.9	C3—N3—Co1	115.9 (3)
N6—C14—C11	113.9 (4)	O3—N3—Co1	122.6 (2)
N6—C14—S1	124.0 (4)	C4—N4—O4	121.7 (3)
C11—C14—S1	122.0 (4)	C4—N4—Co1	116.4 (3)
C16—C15—N6	116.1 (5)	O4—N4—Co1	121.9 (2)
C16—C15—H15A	108.3	C9—N5—C13	118.1 (3)
N6—C15—H15A	108.3	C9—N5—Co1	120.3 (3)
C16—C15—H15B	108.3	C13—N5—Co1	121.6 (3)
N6—C15—H15B	108.3	C14—N6—C15	124.9 (5)
H15A—C15—H15B	107.4	C19—N7—O5	122.6 (4)
C15—C16—C17	119.1 (6)	C19—N7—Co2	115.5 (3)
C15—C16—H16A	107.5	O5—N7—Co2	121.9 (3)
C17—C16—H16A	107.5	C20—N8—O6	122.1 (4)
C15—C16—H16B	107.5	C20—N8—Co2	115.8 (3)
C17—C16—H16B	107.5	O6—N8—Co2	122.1 (3)
H16A—C16—H16B	107.0	C21—N9—O7	120.2 (3)
C18—C17—C16	116.6 (7)	C21—N9—Co2	117.0 (3)
C18—C17—H17A	108.1	O7—N9—Co2	122.8 (3)
C16—C17—H17A	108.1	C22—N10—O8	120.2 (3)
C18—C17—H17B	108.1	C22—N10—Co2	117.2 (3)
C16—C17—H17B	108.1	O8—N10—Co2	122.6 (3)
H17A—C17—H17B	107.3	C27—N11—C31	117.1 (3)
C17—C18—H18A	109.5	C27—N11—Co2	121.2 (2)
C17—C18—H18B	109.5	C31—N11—Co2	121.7 (2)
H18A—C18—H18B	109.5	C32—N12—C33	123.1 (3)
C17—C18—H18C	109.5	N1—O1—H1	101 (3)
H18A—C18—H18C	109.5	N2—O2—H2	106 (3)
H18B—C18—H18C	109.5	N3—O3—H2	105 (2)
N7—C19—C20	113.4 (4)	N4—O4—H1	101 (2)
N7—C19—C23	122.7 (5)	N7—O5—H8	106 (3)
C20—C19—C23	123.9 (5)	N9—O7—H7	102 (4)
N8—C20—C19	113.3 (4)	N10—O8—H8	105 (3)
N8—C20—C24	123.2 (5)	N3—Co1—N4	81.56 (13)
C19—C20—C24	123.5 (4)	N3—Co1—N1	177.86 (13)
N9—C21—C22	112.6 (3)	N4—Co1—N1	99.01 (13)
N9—C21—C25	123.7 (4)	N3—Co1—N2	98.28 (13)
C22—C21—C25	123.7 (4)	N4—Co1—N2	179.43 (13)
N10—C22—C21	112.4 (3)	N1—Co1—N2	81.13 (13)
N10—C22—C26	124.8 (4)	N3—Co1—N5	91.58 (13)
C21—C22—C26	122.7 (4)	N4—Co1—N5	90.24 (12)
C19—C23—H23A	109.5	N1—Co1—N5	90.48 (12)
C19—C23—H23B	109.5	N2—Co1—N5	90.30 (12)
H23A—C23—H23B	109.5	N3—Co1—Cl1	88.31 (10)
C19—C23—H23C	109.5	N4—Co1—Cl1	89.16 (9)
H23A—C23—H23C	109.5	N1—Co1—Cl1	89.63 (9)
H23B—C23—H23C	109.5	N2—Co1—Cl1	90.29 (9)
C20—C24—H24A	109.5	N5—Co1—Cl1	179.40 (9)
C20—C24—H24B	109.5	N7—Co2—N8	81.99 (15)
H24A—C24—H24B	109.5	N7—Co2—N9	179.39 (15)
C20—C24—H24C	109.5	N8—Co2—N9	98.62 (14)
H24A—C24—H24C	109.5	N7—Co2—N10	98.81 (15)
H24B—C24—H24C	109.5	N8—Co2—N10	178.35 (14)
C21—C25—H25A	109.5	N9—Co2—N10	80.58 (13)
C21—C25—H25B	109.5	N7—Co2—N11	89.81 (13)
H25A—C25—H25B	109.5	N8—Co2—N11	90.22 (13)
C21—C25—H25C	109.5	N9—Co2—N11	90.19 (12)
H25A—C25—H25C	109.5	N10—Co2—N11	91.23 (13)
H25B—C25—H25C	109.5	N7—Co2—Cl2	89.78 (10)
C22—C26—H26A	109.5	N8—Co2—Cl2	88.30 (10)
C22—C26—H26B	109.5	N9—Co2—Cl2	90.23 (10)
H26A—C26—H26B	109.5	N10—Co2—Cl2	90.26 (10)
C22—C26—H26C	109.5	N11—Co2—Cl2	178.50 (9)
H26A—C26—H26C	109.5		
			
N1—C1—C2—N2	2.5 (5)	O3—N3—Co1—N5	92.6 (3)
C5—C1—C2—N2	−176.8 (3)	C3—N3—Co1—Cl1	87.6 (3)
N1—C1—C2—C6	−175.7 (3)	O3—N3—Co1—Cl1	−87.9 (3)
C5—C1—C2—C6	4.9 (6)	C4—N4—Co1—N3	0.8 (3)
N3—C3—C4—N4	−1.7 (5)	O4—N4—Co1—N3	179.7 (3)
C7—C3—C4—N4	175.3 (5)	C4—N4—Co1—N1	−177.1 (3)
N3—C3—C4—C8	178.9 (4)	O4—N4—Co1—N1	1.8 (3)
C7—C3—C4—C8	−4.1 (7)	C4—N4—Co1—N2	−73 (15)
N5—C9—C10—C11	0.8 (7)	O4—N4—Co1—N2	106 (15)
C9—C10—C11—C12	−3.1 (7)	C4—N4—Co1—N5	92.3 (3)
C9—C10—C11—C14	176.7 (4)	O4—N4—Co1—N5	−88.7 (3)
C10—C11—C12—C13	2.7 (7)	C4—N4—Co1—Cl1	−87.6 (3)
C14—C11—C12—C13	−177.2 (4)	O4—N4—Co1—Cl1	91.3 (3)
C11—C12—C13—N5	0.2 (7)	C1—N1—Co1—N3	76 (4)
C12—C11—C14—N6	166.6 (5)	O1—N1—Co1—N3	−105 (3)
C10—C11—C14—N6	−13.2 (7)	C1—N1—Co1—N4	−178.7 (3)
C12—C11—C14—S1	−11.3 (6)	O1—N1—Co1—N4	0.6 (3)
C10—C11—C14—S1	168.9 (4)	C1—N1—Co1—N2	1.8 (3)
N6—C15—C16—C17	−51.0 (11)	O1—N1—Co1—N2	−178.8 (3)
C15—C16—C17—C18	−71.2 (9)	C1—N1—Co1—N5	−88.4 (3)
N7—C19—C20—N8	−1.8 (5)	O1—N1—Co1—N5	91.0 (3)
C23—C19—C20—N8	179.4 (4)	C1—N1—Co1—Cl1	92.2 (3)
N7—C19—C20—C24	176.9 (4)	O1—N1—Co1—Cl1	−88.4 (3)
C23—C19—C20—C24	−1.9 (7)	C2—N2—Co1—N3	−178.2 (3)
N9—C21—C22—N10	−3.2 (5)	O2—N2—Co1—N3	0.5 (3)
C25—C21—C22—N10	175.7 (4)	C2—N2—Co1—N4	−105 (15)
N9—C21—C22—C26	173.9 (4)	O2—N2—Co1—N4	74 (15)
C25—C21—C22—C26	−7.2 (6)	C2—N2—Co1—N1	−0.3 (3)
N11—C27—C28—C29	0.7 (6)	O2—N2—Co1—N1	178.4 (3)
C27—C28—C29—C30	−0.4 (5)	C2—N2—Co1—N5	90.1 (3)
C27—C28—C29—C32	−177.7 (3)	O2—N2—Co1—N5	−91.2 (3)
C28—C29—C30—C31	−0.2 (6)	C2—N2—Co1—Cl1	−89.9 (3)
C32—C29—C30—C31	177.1 (4)	O2—N2—Co1—Cl1	88.8 (3)
C29—C30—C31—N11	0.7 (6)	C9—N5—Co1—N3	−42.2 (3)
C30—C29—C32—N12	162.2 (4)	C13—N5—Co1—N3	139.4 (3)
C28—C29—C32—N12	−20.7 (5)	C9—N5—Co1—N4	−123.7 (3)
C30—C29—C32—S2	−17.2 (5)	C13—N5—Co1—N4	57.8 (3)
C28—C29—C32—S2	159.9 (3)	C9—N5—Co1—N1	137.3 (3)
N12—C33—C34—C35	177.7 (4)	C13—N5—Co1—N1	−41.2 (3)
C33—C34—C35—C36	−176.5 (4)	C9—N5—Co1—N2	56.1 (3)
C2—C1—N1—O1	177.8 (3)	C13—N5—Co1—N2	−122.4 (3)
C5—C1—N1—O1	−2.9 (5)	C9—N5—Co1—Cl1	−122 (10)
C2—C1—N1—Co1	−2.8 (4)	C13—N5—Co1—Cl1	59 (10)
C5—C1—N1—Co1	176.5 (3)	C19—N7—Co2—N8	−2.8 (3)
C1—C2—N2—O2	−179.8 (3)	O5—N7—Co2—N8	177.8 (3)
C6—C2—N2—O2	−1.6 (5)	C19—N7—Co2—N9	178 (100)
C1—C2—N2—Co1	−1.1 (4)	O5—N7—Co2—N9	−2(14)
C6—C2—N2—Co1	177.1 (3)	C19—N7—Co2—N10	178.6 (3)
C4—C3—N3—O3	178.0 (3)	O5—N7—Co2—N10	−0.8 (3)
C7—C3—N3—O3	0.9 (7)	C19—N7—Co2—N11	87.4 (3)
C4—C3—N3—Co1	2.4 (5)	O5—N7—Co2—N11	−92.0 (3)
C7—C3—N3—Co1	−174.7 (4)	C19—N7—Co2—Cl2	−91.2 (3)
C3—C4—N4—O4	−178.7 (3)	O5—N7—Co2—Cl2	89.5 (3)
C8—C4—N4—O4	0.6 (6)	C20—N8—Co2—N7	1.8 (3)
C3—C4—N4—Co1	0.3 (4)	O6—N8—Co2—N7	−176.4 (3)
C8—C4—N4—Co1	179.6 (3)	C20—N8—Co2—N9	−178.2 (3)
C10—C9—N5—C13	2.1 (7)	O6—N8—Co2—N9	3.6 (3)
C10—C9—N5—Co1	−176.4 (4)	C20—N8—Co2—N10	121 (5)
C12—C13—N5—C9	−2.7 (6)	O6—N8—Co2—N10	−57 (5)
C12—C13—N5—Co1	175.9 (3)	C20—N8—Co2—N11	−88.0 (3)
C11—C14—N6—C15	−172.2 (5)	O6—N8—Co2—N11	93.8 (3)
S1—C14—N6—C15	5.6 (8)	C20—N8—Co2—Cl2	91.8 (3)
C16—C15—N6—C14	−86.9 (8)	O6—N8—Co2—Cl2	−86.4 (3)
C20—C19—N7—O5	−177.4 (3)	C21—N9—Co2—N7	−3(14)
C23—C19—N7—O5	1.4 (6)	O7—N9—Co2—N7	−180 (100)
C20—C19—N7—Co2	3.3 (4)	C21—N9—Co2—N8	178.0 (3)
C23—C19—N7—Co2	−177.9 (3)	O7—N9—Co2—N8	1.0 (3)
C19—C20—N8—O6	177.7 (3)	C21—N9—Co2—N10	−3.4 (3)
C24—C20—N8—O6	−0.9 (6)	O7—N9—Co2—N10	179.6 (3)
C19—C20—N8—Co2	−0.6 (4)	C21—N9—Co2—N11	87.8 (3)
C24—C20—N8—Co2	−179.2 (3)	O7—N9—Co2—N11	−89.2 (3)
C22—C21—N9—O7	−178.4 (3)	C21—N9—Co2—Cl2	−93.6 (3)
C25—C21—N9—O7	2.7 (6)	O7—N9—Co2—Cl2	89.3 (3)
C22—C21—N9—Co2	4.5 (4)	C22—N10—Co2—N7	−178.5 (3)
C25—C21—N9—Co2	−174.4 (3)	O8—N10—Co2—N7	3.6 (3)
C21—C22—N10—O8	178.3 (3)	C22—N10—Co2—N8	63 (5)
C26—C22—N10—O8	1.4 (6)	O8—N10—Co2—N8	−115 (5)
C21—C22—N10—Co2	0.4 (4)	C22—N10—Co2—N9	1.5 (3)
C26—C22—N10—Co2	−176.5 (3)	O8—N10—Co2—N9	−176.4 (3)
C28—C27—N11—C31	−0.2 (5)	C22—N10—Co2—N11	−88.5 (3)
C28—C27—N11—Co2	−179.9 (3)	O8—N10—Co2—N11	93.6 (3)
C30—C31—N11—C27	−0.4 (6)	C22—N10—Co2—Cl2	91.7 (3)
C30—C31—N11—Co2	179.2 (3)	O8—N10—Co2—Cl2	−86.2 (3)
C29—C32—N12—C33	175.5 (3)	C27—N11—Co2—N7	52.7 (3)
S2—C32—N12—C33	−5.2 (6)	C31—N11—Co2—N7	−126.9 (3)
C34—C33—N12—C32	−78.5 (5)	C27—N11—Co2—N8	134.7 (3)
C3—N3—Co1—N4	−1.8 (3)	C31—N11—Co2—N8	−44.9 (3)
O3—N3—Co1—N4	−177.4 (3)	C27—N11—Co2—N9	−126.7 (3)
C3—N3—Co1—N1	104 (3)	C31—N11—Co2—N9	53.7 (3)
O3—N3—Co1—N1	−72 (4)	C27—N11—Co2—N10	−46.1 (3)
C3—N3—Co1—N2	177.6 (3)	C31—N11—Co2—N10	134.3 (3)
O3—N3—Co1—N2	2.1 (3)	C27—N11—Co2—Cl2	127 (3)
C3—N3—Co1—N5	−91.8 (3)	C31—N11—Co2—Cl2	−52 (4)

### Hydrogen-bond geometry (Å, °)

**Table:**

*D*—H···*A*	*D*—H	H···*A*	*D*···*A*	*D*—H···*A*
O1—H1···O4	1.20 (6)	1.32 (6)	2.491 (4)	162 (5)
O2—H2···O3	1.12 (6)	1.39 (6)	2.469 (4)	158 (5)
O8—H8···O5	1.17 (8)	1.34 (8)	2.486 (5)	165 (6)
O7—H7···O6	0.98 (7)	1.52 (7)	2.490 (5)	171 (7)

## References

[bb13] Altomare, A., Cascarano, G., Giacovazzo, C. & Guagliardi, A. (1993). *J. Appl. Cryst.* **26**, 343–350.

[bb1] Bresciani-Pahor, N., Forcolin, M., Marzilli, L. G., Randaccio, L., Summers, M. F. & Toscano, P. J. (1985). *Coord. Chem. Rev.* **63**, 1–125.

[bb2] Bruker (2004). *APEX2*, *SAINT* and *SADABS* Bruker AXS Inc., Madison, Wisconsin, USA.

[bb3] Dolphin, D. (1982). Editor. *B_12_*, Vols. 1 and 2. New York: Wiley.

[bb4] Englert, U., Heger, G., Kummerle, E. & Wang, R. (1999). *Z. Kristallogr.* **214**, 71–74

[bb5] Englert, U., Kirch, M. & Knur, N. (2000). *Z. Kristallogr.* **215**, 260–263.

[bb6] Geno, M. K. & Halpern, J. (1987). *J. Am. Chem. Soc.* 109, 1238–1240.

[bb7] Kavitha, T., Revathi, C., Hemalatha, M., Dayalan, A. & Ponnuswamy, M. N. (2008). *Acta Cryst.* E**64**, o114. 10.1107/S1600536807062125 PMC2915185 21200678

[bb8] Ramesh, P., SubbiahPandi, A., Jothi, P., Revathi, C. & Dayalan, A. (2008). *Acta Cryst.* E**64**, m300–m301. 10.1107/S1600536807068407 PMC2960257 21201276

[bb9] Schrauzer, G. N. & Kohnel, K. (1964). *Chem. Ber.* **97**, 3056.

[bb10] Sheldrick, G. M. (2008). *Acta Cryst.* A**64**, 112–122. 10.1107/S0108767307043930 18156677

[bb11] Spek, A. L. (2009). *Acta Cryst.* D**65**, 148–155. 10.1107/S090744490804362X PMC2631630 19171970

[bb12] Trogler, W. C., Stewart, R. C., Epps, L. A. & Marzilli, L. G. (1974). *Inorg. Chem.* **13**, 1564–1569.

